# STAT4 Mediates IL-6 Trans-Signaling Arrhythmias in High Fat Diet Guinea Pig Heart

**DOI:** 10.3390/ijms25147813

**Published:** 2024-07-17

**Authors:** Andrea Corbin, Kelly A. Aromolaran, Ademuyiwa S. Aromolaran

**Affiliations:** 1 Nora Eccles Harrison Cardiovascular Research and Training Institute (CVRTI), University of Utah School of Medicine, Salt Lake City, UT 84132, USA; andrea.corbin@utah.edu (A.C.); kelly.aromolaran@utah.edu (K.A.A.); 2 Department of Biomedical Engineering, University of Utah School of Medicine, Salt Lake City, UT 84132, USA; 3 Department of Surgery, Division of Cardiothoracic Surgery, Nutrition & Integrative Physiology, Biochemistry & Molecular Medicine Program, University of Utah School of Medicine, Salt Lake City, UT 84132, USA

**Keywords:** signal transducer and activator of transcription 4, interleukin-6 trans-signaling, guinea pig, arrhythmias

## Abstract

Obesity is a major risk factor for the development of life-threatening malignant ventricular tachyarrhythmias (VT) and sudden cardiac death (SCD). Risks may be highest for patients with high levels of the proinflammatory cytokine interleukin (IL)-6. We used our guinea pig model of high-fat diet (HFD)-induced arrhythmias that exhibit a heightened proinflammatory-like pathology, which is also observed in human obesity arrhythmias, as well as immunofluorescence and confocal microscopy approaches to evaluate the pathological IL-6 trans-signaling function and explore the underlying mechanisms. Using blind-stick and electrocardiogram (ECG) techniques, we tested the hypothesis that heightened IL-6 trans-signaling would exhibit increased ventricular arrhythmia/SCD incidence and underlying arrhythmia substrates. Remarkably, compared to low-fat diet (LFD)-fed controls, HFD promoted phosphorylation of the IL-6 signal transducer and activator of transcription 4 (STAT4), leading to its activation and enhanced nuclear translocation of pSTAT4/STAT4 compared to LFD controls and pSTAT3/STAT3 nuclear expression. Overactivation of IL-6 trans-signaling in guinea pigs prolonged the QT interval, which resulted in greater susceptibility to arrhythmias/SCD with isoproterenol challenge, as also observed with the downstream Janus kinase (JAK) 2 activator. These findings may have potentially profound implications for more effective arrhythmia therapy in the vulnerable obese patient population.

## 1. Introduction

Ventricular tachycardia (VT) accounts for the electrophysiological events leading to sudden cardiac death (SCD) in 60–80% of pathologies [1]. At the cellular level, pathological modulation of the expression and/or electrophysiological function of major cardiac ion channel subunits, with a subsequent prolongation of the ventricular action potential duration, underlies an elevated risk of prolonged QT interval linked life-threatening VT [2,3]. Obesity increases VT/SCD risk, particularly under conditions of repolarization disorder and prolonged QT interval [4]. The pathology of obesity-related arrhythmias is associated with abnormal accumulation of lipids (lipotoxicity), which induces an increase in proinflammatory cytokines, including IL-6, elevating the risk of adverse ventricular electrical remodeling [5]. Preventing such lipotoxic effects is a promising direction for therapeutic intervention into the progression of VT, and ultimately prevention of SCD.

IL-6 is a powerful predictor of the severity of heart disease [6]. Classical IL-6 signaling occurs through the membrane-bound receptor (IL-6Rα)- glycoprotein 130 (gp130 receptor complex) and mediates homeostasis and regenerative functions [7]. The soluble IL-6 receptor (sIL-6R) is generated by extracellular shedding or alternative processing of the mRNA encoding the IL-6R. IL-6 proinflammatory effects are mediated via trans-signaling, whereby IL-6 binds to the sIL-6R, leading to activation of the downstream Janus kinase (JAK)-signal transducer and activator of transcription (STAT) pathway [7]. STATs, upon phosphorylation, dimerize and translocate to the nucleus, where they can induce the expression of genes involved in proliferation and differentiation. While there is overwhelming evidence supporting the presence of inflammation in heart disease and proinflammatory cytokine channelopathies [5,8], there are still unanswered questions in the arrhythmia field regarding whether/how IL-6 trans-signaling remodels ventricular pathology and VT predisposition in obese heart and the molecular mechanisms involved.

## 2. Results

### 2.1. Effect of HFD on Cardiac Fibrosis in Guinea Pig

**Figure 1:**
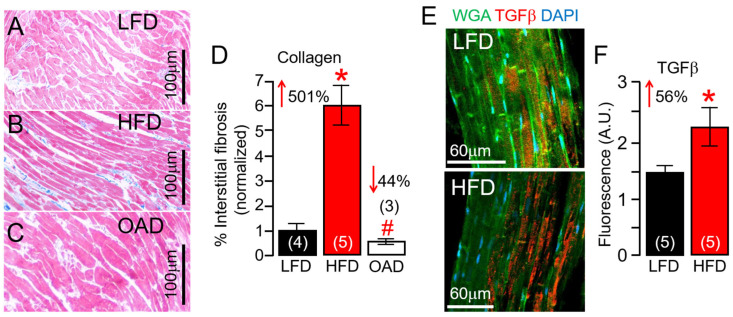
**Effects of high-fat diet feeding on guinea pig hearts.** Compared to LFD-fed male and female guinea pig ventricular samples (**A**), HFD animals showed a marked increase in interstitial fibrosis (**B**), but not in OAD-fed guinea pigs (**C**). (**D**) Quantification of percent (%) fibrotic area in the ventricular samples (*n* = 4 LFD, 5 HFD, 3 OAD). (**E**) Confocal immunofluorescent images of LFD (**Top image**) and HFD (**Bottom image**) guinea pig ventricular slices analyzed using TGF-β antibodies (Red), WGA (Green), and DAPI (Blue), showed significantly increased TGF-β fluorescence labeling in HFD ventricular tissue slices compared to LFD controls (**F**), suggesting HFD feeding promotes the overactivation of inflammatory signaling in guinea pig heart (*n* = 5 LFD, 5 HFD). Numbers in brackets represent the number of hearts examined. Data columns represent mean ± S.E.M, *^,#^ Statistical significance at *p* < 0.05.

Male and female guinea pigs were challenged, ad libitum, for 14 weeks with LFD and HFD that we previously showed promoted significant changes in total cholesterol and total triglycerides [9]. Compared to LFD, HFD feeding increased the change (Δ) in body weight (ΔBW) (33 or 402.4 ± 57.6 g, *n* = 8; vs. 435.6 ± 34 g; *n* = 15, *p* = 0.62). In other experiments, ΔBW was Δ34.6 (or 402.4 ± 57.6 g, *n* = 8; vs. 437.05 ± 47.5 g; *n* = 8, *p* = 0.65) and Δ (-) 1.47 for the OAD challenged group when compared to the LFD and HFD guinea pigs, respectively. The HFD group showed a marked increase in % interstitial fibrosis in guinea pig ventricles (Figure 1A,B), and this could be reduced to LFD control levels with OAD feeding (Figure 1C). After 14 weeks, % fibrosis was increased by 501% in the HFD group, but the extent of fibrosis in the OAD group was similar to LFD-fed controls (Figure 1D). Our data show a significant increase (from 1% to about 6%) in interstitial fibrosis in HFD hearts when compared to LFD-challenged controls. However, while this is a large increase in the amount of interstitial fibrosis, it is not severe.

Furthermore, confocal imaging of LFD and HFD ventricular tissue was used to determine whether the transforming growth factor beta (TGF-β) pathway expression, known to be profibrotic, was affected. In five separate experiments using HFD guinea pig ventricular tissue, TGF-β protein expression was significantly increased (by 56%, Figure 1E,F) compared to the LFD controls. These data demonstrated the fibrosis-promoting effect of chronic HFD feeding in guinea pigs and that OAD may be associated with blunted % fibrosis in the heart, which could lead to beneficial ventricular remodeling and reduced risk of arrhythmias.

### 2.2. HFD Feeding Is Associated with Upregulated Cardiac IL-6 Linked Inflammation in Guinea Pig Ventricular Tissue

**Figure 2:**
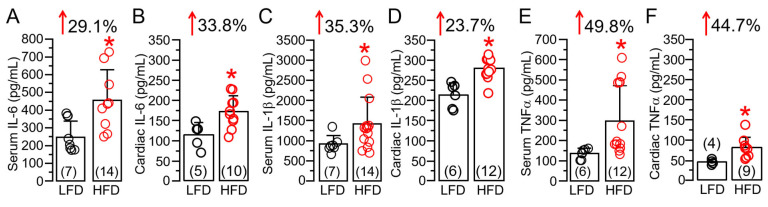
**High-fat diet feeding is associated with overactivation of proinflammatory cytokine expression in guinea pigs.** Column graphs represent ELISA assay quantification (in pg/mL), of IL-6 (**A**,**B**), IL-1β (**C**,**D**), TNF-α (**E**,**F**) measured in LFD and HFD guinea pig serum and ventricular samples, respectively. Comparison is made between serum vs. cardiac levels for a specific cytokine. Data columns are mean ± S.E.M, *n* = 4–7 LFD, 9–14 HFD. * Statistical significance at *p* < 0.05.

There have been previous reports of an increased release of the immunomodulatory proinflammatory cytokine IL-6 in obese patients [10,11,12], leading to a state of chronic inflammation [7,13,14]. We also measured cardiac (or local) and systemic IL-6 levels following HFD feeding. Our data revealed that HFD-challenged guinea pigs showed a significant increase in serum (Figure 2A) and cardiac tissue (Figure 2B) IL-6 levels. On average, serum and ventricular tissue IL-6 was significantly (* *p* < 0.05) increased, by 29.1% (Figure 2A) and 33.8% (Figure 2B), respectively, in HFD-fed guinea pigs compared to LFD controls. A similar picture emerged with IL-1β (Figure 2C,D, 35.3% vs. 23.7%) and TNF-α (Figure 2E,F, 49.8% vs. 44.7%), further establishing our HFD guinea pig as an inflammatory model. Moreover, compared to serum levels, HFD induced a larger increase in local cardiac IL-6 but this difference was not seen with IL-1β and TNF-α, suggesting that the overactivated IL-6 signaling pathway may elicit a greater inflammatory response and exacerbate cardiac dysfunction.

### 2.3. Overactivated IL-6 Trans-Signaling Causes Pathological Guinea Pig Heart Electrophysiology and Increased Arrhythmic Risk

**Figure 3:**
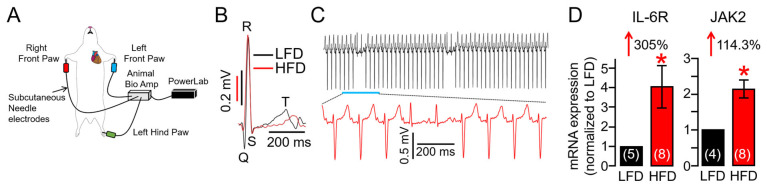
**High-fat diet adult guinea pigs show vulnerability to spontaneous ventricular arrhythmogenesis.** (**A**) Cartoon depiction of the experimental protocol used to assess the effect of HFD feeding on QT interval and VT risk. (**B**) Overlay of representative traces of surface ECG recorded in LFD (black trace) and HFD (red trace), showing that HFD guinea pigs displayed prolonged QT interval compared to LFD controls. (**C**) Exemplar ECG traces demonstrating triggered ventricular arrhythmic signatures and arrhythmias measured in HFD-challenged adult guinea pigs. (**D**) Averaged mRNA expression of IL-6R and JAK2 measured in LFD and HFD ventricular tissue samples (*n* = 4–5 LFD, 8 HFD). Data are expressed as the fold change in IL-6R and JAK2 expression compared with LFD after normalization to GAPDH. Data revealed HFD feeding induced profound increases in IL-6R and JAK2 expression compared to LFD-fed controls demonstrating overactivation of IL-6 trans-signaling in HFD hearts. Data columns are mean ± S.E.M, *n* = 4–8 separate experiments. * Statistical significance at *p* < 0.05.

Obesity increases VT risk [15,16,17,18], particularly under conditions of repolarization disorder and prolonged heart rate corrected QT interval (QT_c_, an established risk factor for VT) [4,19,20]. We discovered, in the surface electrocardiogram (Figure 3A), that HFD feeding is accompanied by a prolonged QT_c_ interval (Figure 3B, Table 1), and this can be prevented with OAD feeding (Table 2). We further found that HFD guinea pigs displayed increased risks of spontaneous ventricular arrhythmias (sinus rhythm with abnormal conduction in the ventricular conduction system leading to altered QRS morphology and T waves suggesting bundle branch block. Figure 3C) compared to LFD-fed controls, consistent with increased risk markers of VT/SCD in HFD-linked obesity. Given the established role of heightened IL-6 signaling in obesity, endogenous IL-6R and JAK2 transcripts, which are part of the pro-inflammatory IL-6 pathway, were next assessed in LFD and HFD hearts using qRT-PCR assays. Compared to LFD, our data revealed that HFD guinea pigs had increased expression levels of ventricular IL-6R and JAK2 genes (Figure 3D), substantiating our observation of HFD-linked overactivation of IL-6 signaling (Figure 2).

**Figure 4:**
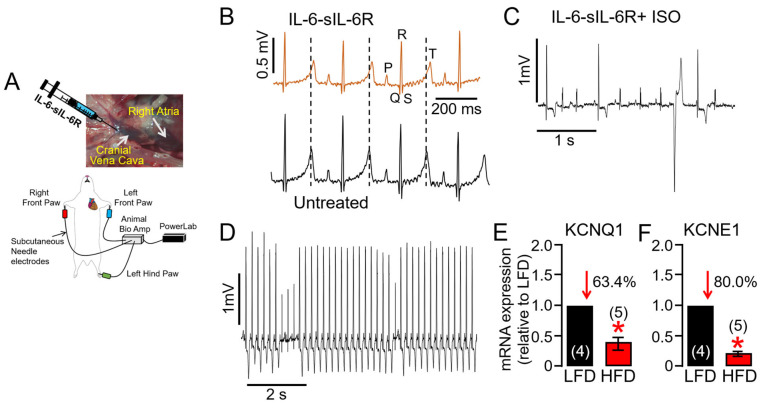
**Overstimulation of IL-6 trans-signaling induces QT prolongation in guinea pigs under conditions of cardiac challenge.** (**A**) Experimental protocol used to assess the effect of the overstimulation of IL-6 trans-signaling on guinea pig QT interval induced by recombinant IL-6 and sIL-6R injected into the cranial vena cava (CVC). (**B**) Representative traces of surface ECG recorded in IL-6-sIL-6R (**Top panel**) and untreated control (**Bottom panel**) guinea pigs. The subsequent exposure to the β-adrenergic receptor agonist ISO in the continued presence of IL-6-sIL-6R triggered ventricular arrhythmias (**C**,**D**). Averaged mRNA expression of KCNQ1 (**E**) and KCNE1 (**F**) measured in LFD and HFD ventricular tissue samples (*n* = 4 LFD, 5 HFD). Data are expressed as the fold change in expression compared with LFD after normalization to GAPDH. Our data revealed severely reduced expression of KCNQ1 and KCNE1 coding for *I_Ks_* in HFD ventricular samples, in agreement with dramatic ventricular electrical remodeling and increased risk of exercise-induced VT. Data columns represent mean ± S.E.M from *n* = 4–5 guinea pig/group, * Statistical significance at *p* < 0.05.

To test the hypothesis that increased IL-6 levels in obesity directly contribute to an increased risk of VT/SCD and may represent a novel therapeutic target, adult guinea pigs were exposed to activators of IL-6 trans-signaling (recombinant IL-6-sIL-6R) through the cranial vena cava and right atrium (Figure 4A, as we previously [8] established), and then changes in QT_c_ and susceptibility to ventricular arrhythmias were monitored. Figure 4B shows typical ECG traces measured in guinea pigs exposed to IL-6-sIL-6R. Compared to vehicle-injected controls, QT_c_ and change in QT_c_ interval (ΔQT_c_) were significantly greater (Table 2) in IL-6-sIL-6R guinea pigs. To further uncover overactivated IL-6 trans-signaling-linked electrophysiological defects, we performed a provocative test with isoproterenol (ISO, 0.5 mg/kg) to reproduce sympathetic stimulation. Shown in Figure 4C,D are examples of ventricular arrhythmias induced by an ISO challenge in IL-6-sIL-6R guinea pigs. On average, IL-6-sIL-6R-ISO challenged guinea pigs showed a higher total occurrence of arrhythmias (Figure 4C,D 5/5/100% guinea pigs) and ultimately SCD (2/5/40% guinea pigs) compared to controls (1/4/25%, and 0/4/0% respectively), consistent with the signature high-risk proarrhythmic effect that underlies fatal arrhythmias of ventricular origin in patients. Compared to LFD-fed guinea pig ventricles, HFD severely depressed the expression of genes encoding for KCNQ1 (Figure 4E) and KCNE1 (Figure 4F) channel subunits, consistent with the ability of *I_Ks_* to limit action potential duration (APD) during sympathetic stimulation and its potential involvement in VT/SCD risk in obese patients with heightened inflammatory response, particularly during exercise [17].

**Figure 5:**
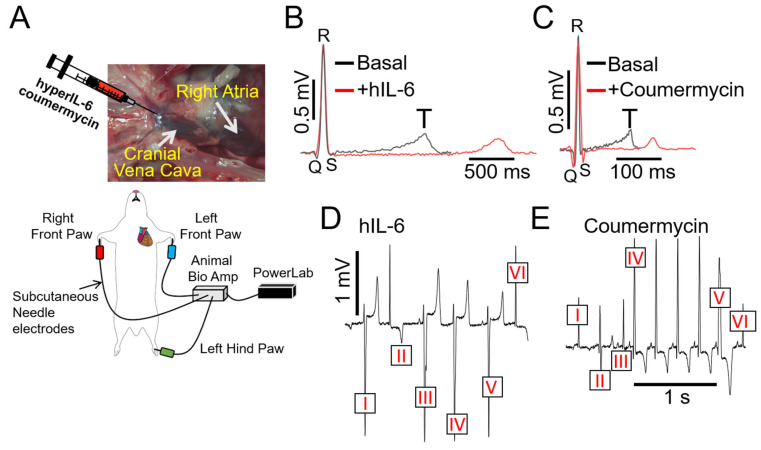
**Direct stimulation of IL-6 trans-signaling with hIL-6 and coumermycin induces severe arrhythmias in guinea pigs.** (**A**) Cartoon illustration of experimental protocol and arrhythmia inducibility in guinea pigs. Guinea pigs challenged with hIL-6 (**B**) or JAK2 activator (**C**) severely prolonged the QT interval and triggered ventricular arrhythmic signatures (**D**,**E**).

As a complementary approach, guinea pigs were challenged with designer hIL-6 [21,22,23,24] (25 ng/mL/kg), a highly potent and selective (independent of classic signaling) activator of IL-6 trans-signaling. Under baseline conditions, ECGs were recorded from all fifteen initially unchallenged guinea pigs, which served as their own controls (Figure 5A). On average, hIL-6 (Figure 5B) alone or the JAK2 agonist coumermycin (C-A1 [25], 100 μg/kg, Figure 5C) significantly (* *p* < 0.05) prolonged QT_c_ interval and profoundly increased ΔQT_c_ (Table 2, or by Δ34.8 ± 8.15 ms and Δ38.4 ± 10.9 ms, respectively, vs. Δ21.66 ± 6.12 ms with IL-6-sIL-6R), compared to guinea pigs challenged with the first natural and specific inhibitor of IL-6 trans-signaling, olamkicept (2.5 mg/kg [26], Table 2), or to saline control. We also discovered that hIL-6 or coumermycin directly triggered arrhythmias (Figure 5D,E). For example, in Figure 5D, ventricular complexes III and V are preceded by a P-wave, but with inconsistent PR intervals. Furthermore, ventricular complexes I and IV have a similar shape as III and V, but with no visible P-wave, suggesting complexes of ventricular origin. Complexes II and VI display a narrow QRS complex, but without a P-wave consistent with arrhythmias of ventricular origin, likely from the conduction system. Furthermore, in Figure 5E, the complex V is preceded by a P-wave, but the PR interval is shorter than the four previous beats. In addition, the QRS is wider in complex V, reminiscent of a premature ventricular beat (or premature ventricular complex, PVC). Complex VI is preceded by a P wave, and the PR interval seems to be similar to complex IV and suggests a sinus beat. Similarly, complexes I and III are similar to VI, which could mean those are also sinus beats. Complex II is different and could be a ventricular premature beat, likely originating from the conduction system. Taken together, our data suggest that IL-6 trans-signaling may mediate inflammation ventricular arrhythmogenesis, in part via IL-6R/JAK2 overactivation.

### 2.4. IL-18 Enhances IL-6 Trans-Signaling Effects on Guinea Pig Ventricular Cardiac Electrophysiology

**Figure 6:**
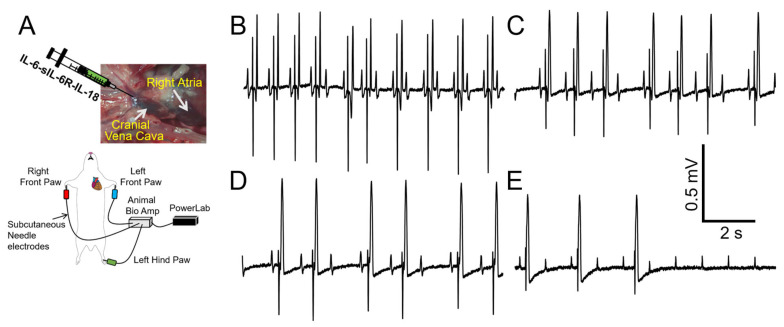
**IL-18 treatment increases the risk of severe arrhythmia under conditions of overactive IL-6 trans-signaling.** (**A**) Cartoon illustration of experimental protocol and arrhythmia inducibility in conditions of cardiac hyperinflammation. IL-6-sIL-6R with IL-18 displayed conduction block and asystole (**B**–**E**) in 4/6 guinea pigs, in-line with a potential role for IL-18 in exacerbating dramatic ventricular electrical activity (ion channel remodeling, action potential phenotypes), due to overactive IL-6 trans-signaling.

Despite the advantages of selectively investigating a role for IL-6 trans-signaling in obesity-linked VT, our data (Figure 5) revealed that while overactive IL-6-trans-signaling ΔQT_c_ was statistically significant, it was less remarkable than the effects of HFD, suggesting that other cytokines (IL-1β, TNF-α, IL-18, Figure 2) likely contributed to the HFD-linked ΔQT_c_. While the effects of IL-1β and TNF-α on cardiac electrophysiology have been studied [5,27], the role of IL-18 is under-explored. IL-18 has increased expression in obesity [28,29,30] and can induce the production of copious amounts of IL-6, serving as an amplifier of IL-6 effects. We hypothesized that IL-18 may heighten IL-6 effects on ventricular electrophysiology and arrhythmia risks. To test this hypothesis, we challenged guinea pigs with a combination of recombinant IL-6-sIL-6R and IL-18 (10 μg/kg [31,32,33]). Table 2 shows that IL-18 significantly increased QT_c_ interval due to IL-6-sIL-6R. Furthermore, in the presence of IL-18, IL-6-sIL-6 guinea pigs displayed first degree atrioventricular (AV) block that transitioned into complete AV dissociation in 4/6 guinea pigs (Figure 6). Panel B shows 2:1 AV block (with a distinct T-wave after the pause). Panels C and D show Wenckebach AV block (prolong PR interval until AV conduction fails) with a shortened QT interval, as well as the shape of a T-wave. Panel E shows complete AV block (second half of the trace). Our data suggests a potential role for IL-18 in IL-6 trans-signaling-linked pathological ion channel remodeling, action potential phenotypes, and increased propensity for heart block.

### 2.5. Proinflammatory Cytokines Induce Dramatic Ventricular Electrophysiology Remodeling during Adverse Sympathetic Regulation and Are Associated with VT in Guinea Pig

**Figure 7:**
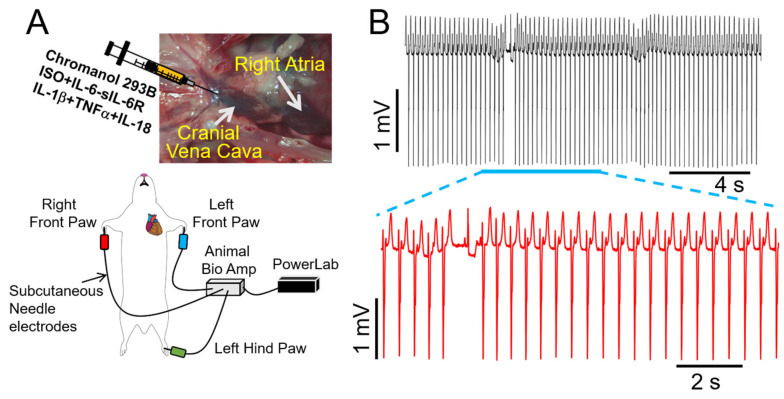
**Effect of multiple proinflammatory cytokines on arrhythmia risk in guinea pigs under conditions of *I_Ks_* inhibition.** (**A**) Cartoon illustration of experimental protocol for arrhythmia induction. (**B**) Exemplar ECG traces recorded from guinea pigs initially treated with the *I_Ks_* blocker chromanol 293B, and subsequently challenged with cytomix (IL-6-sIL-6R + IL-1β + TNF-α + IL-18) and ISO. Expanded view of ventricular electrical activities revealed severe arrhythmogenesis. Data were generated from three different guinea pig hearts.

During high β-adrenergic activity, *I_Ks_* becomes larger and displays faster activation kinetics [34]. Thus, *I_Ks_* becomes critical for ventricular action potential shortening [35] during tachycardia. Therefore, *I_Ks_* is another key component of the “repolarization reserve” in the ventricles [36], suggesting that impaired *I_Ks_* function would be expected to be proarrhythmic in the setting of chronically elevated key obesity components (sympathetic activation, heightened proinflammatory cytokine levels). In our study, we observed a significant prolongation of QT_c_ and ΔQT_c_ in guinea pigs treated with the chromanol 293B (2 mg/kg), an *I_Ks_* blocker [37], as compared with untreated guinea pigs (Table 2). Moreover, a similar picture emerged in chromanol 293B guinea pigs subsequently exposed to ISO alone (10 min) or with a cytomix (containing IL-6-sIL-6R + IL-1β + TNF-α + IL-18***,*** 10–30 min, Table 2). Under these conditions, we further observed severe induction of ventricular arrhythmias, which lasted for ~20 s (Figure 7A,B), suggesting that reductions in *I_Ks_* may exacerbate ventricular electrical remodeling and contribute to exercise-induced VT in obese conditions.

### 2.6. ERG1a Protein Expression Is Reduced in HFD Hearts

**Figure 8:**
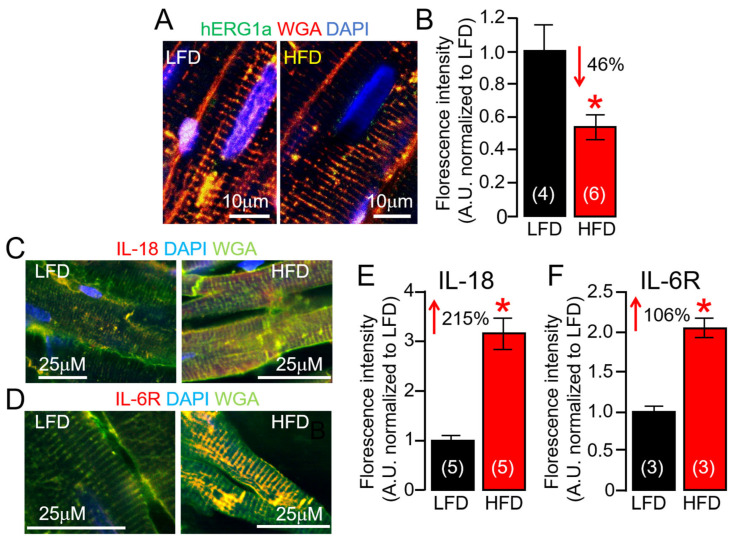
**Effects of high-fat diet on subcellular localization of ERG1a channels, IL-18, and IL-6R in guinea pig ventricular tissue slices.** (**A**) Confocal images of permeabilized LFD and HFD adult guinea pig ventricular tissue samples showing ERG1a (Green), WGA (Red), and DAPI (Blue) fluorescence images. (**B**) Normalized mean fluorescence signals from yellow positive tissue samples provide an index of relative surface density of ERG1a in HFD samples (*n* = 4 LFD, 6 HFD). Data for IL-18 (**C**) or IL-6R (**D**) antibodies indicate increased surface expression of IL-18 (**E**) and IL-6R (**F**) in HFD compared to LFD-fed controls, same format as (**A**,**B**) (*n* = 3–5 LFD, 3–5 HFD). Data columns are mean ± S.E.M. ERG1a experiments had 4–8 images per animal. IL-18 and IL-6R experiments had 3–4 images and 3–7 images per animal, respectively. * Statistical significance at *p* < 0.05.

Ion channel dysfunction remains one of the crucial factors in VT initiation, and in most cases of acquired arrhythmias, *I_Kr_* remains the dominant channel of clinical cardiotoxicity concern; and the FDA and European Medicines Agency requires screening against the human ether-à-go-go-related gene (hERG) for all new drugs being evaluated [38,39]. At the cellular level, decreases in *I_Kr_* function underlies an elevated risk for VT [8]. Next, we hypothesized that ERG1 subunit expression changes in response to HFD feeding may occur at the posttranslational level. To test this hypothesis, we assessed surface ERG1a protein expression using confocal imaging of ventricular slices (Figure 8). We found that ERG1a membrane expression was severely reduced in HFD ventricular slices compared to LFD controls (Figure 8A,B). Thus, we investigated if HFD altered IL-18 and IL-6R protein expression as a readout for a ventricular inflammatory state and found that it did. HFD guinea pig ventricular slices revealed that both IL-18 (Figure 8C) and IL-6R (Figure 8D) protein expressions were more significantly upregulated than LFD-fed controls. Furthermore, IL-18 slices displayed greater fluorescence (215%, Figure 8E) labelling of the sarcolemma and t-tubules in HFD compared to IL-6R (106%, Figure 8F). Together, our data suggest the effects of IL-18 on IL-6 trans-signaling linked QT_c_ (Table 2) could be the result of severe depression of *I_Kr_*, likely due to enhanced defective ERG channel subunit functional expression at the cardiomyocyte cell-surface.

### 2.7. HFD Feeding Is Associated with Increased STAT4 Expression in Guinea Pig Heart

**Figure 9:**
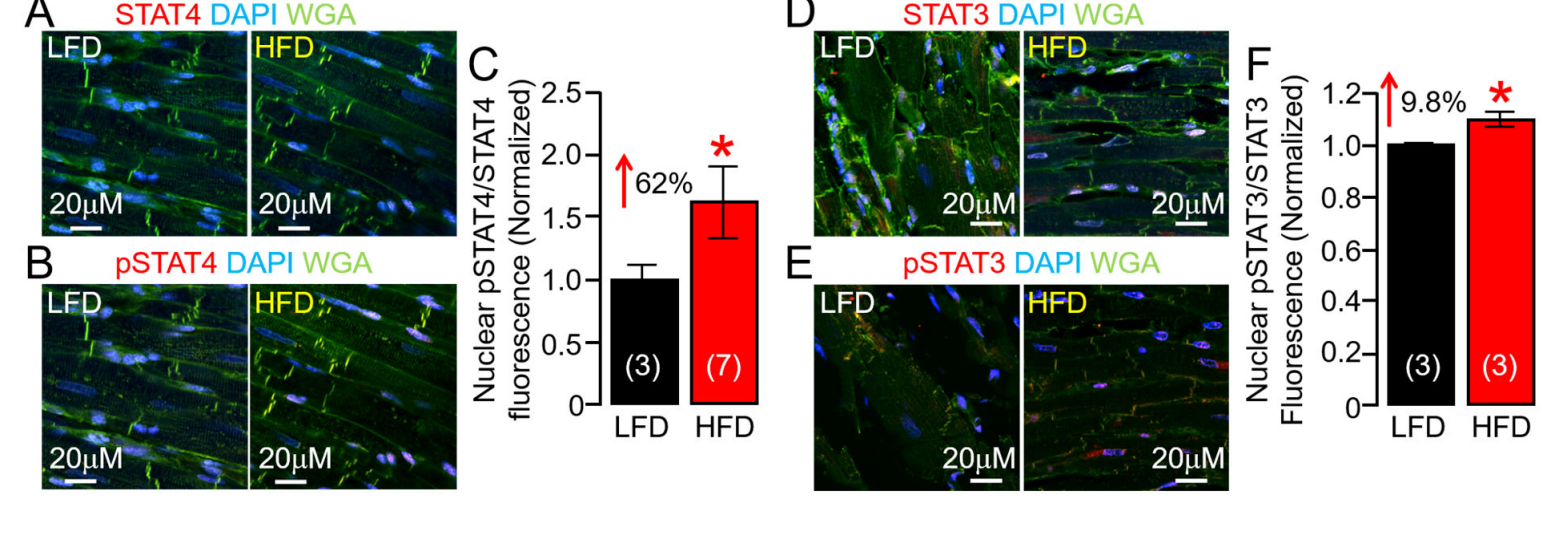
**High-fat diet selectively induces the phosphorylation of STAT4 in guinea pig ventricular tissue slices.** Confocal images of permeabilized LFD and HFD adult guinea pig ventricular tissue samples showing STAT4 (**A**) or pSTAT4 ((**B**), Red), WGA (Green), and DAPI (Blue) fluorescence images (**A**,**B**). (**C**) Normalized mean fluorescence signals from magenta- and magenta-blue positive tissue ventricular samples provide an index of relative expression of pSTAT4/STAT4 and nuclear translocation (*n* = 3 LFD, 7 HFD). HFD promotes phosphorylation of STAT4, leading to its activation and enhanced nuclear translocation of pSTAT4/STAT4 compared to LFD controls and pSTAT3/STAT3 nuclear expression. (**D**–**F**) Data for STAT3 (**D**) and pSTAT3 (**E**), same format as (**A**–**C**) (*n* = 3 LFD, 3 HFD). Data columns are mean ± S.E.M. Three images are included for each animal, with the total number of segmented nuclei per image ranging from 8 to 24. * Statistical significance at *p* < 0.05.

IL-6 proinflammatory effects are mediated via trans-signaling, whereby IL-6 binds to the sIL-6R [40,41] and engages gp130 on target cells, leading to activation of downstream JAK-STAT [40,42,43,44]. STATs, upon phosphorylation, dimerize and translocate to the nucleus, where they can induce the expression of genes involved in proliferation and differentiation [45,46,47,48]. Although there is evidence that STAT4 is involved in cardiovascular diseases [49,50,51], the potential role of STAT4 in obesity VT risk is unknown. We investigated whether STAT4 was functionally active in our HFD guinea pig model. In agreement with overactive IL-6 and JAK2 activity, our data revealed, for the first time, that HFD increased phosphorylation of STAT4, consistent with enhanced inflammation, leading to its activation and enhanced nuclear translocation of pSTAT4/STAT4 compared to LFD control ventricular tissue slices (Figure 9A–C), and pSTAT3/STAT3 nuclear expression (Figure 9D–F), suggesting a minimal role for activated STAT3 in our HFD model. Our results demonstrated that HFD-induced inflammation increases cardiac STAT4 activity, supporting the pathological role of IL-6 trans-signaling in the obese heart.

### 2.8. Lipotoxicity Promotes Overactivation of IL-6 Trans-Signaling in Guinea Pig Ventricular Myocytes via Lipid Droplet Accumulation

**Figure 10:**
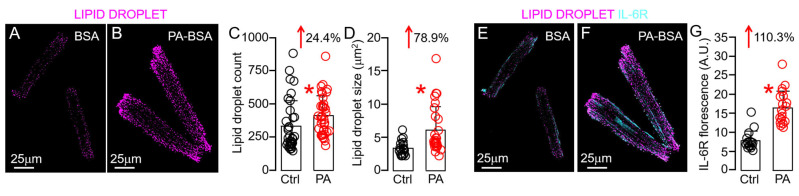
**Lipotoxicity induces lipid droplet remodeling and increased expression of IL-6R in guinea pig ventricular tissue slices**. Confocal images of permeabilized adult guinea pig ventricular cardiomyocytes showing lipid droplet (magenta) accumulation in untreated (**A**) and PA-BSA ((**B**) a potent inducer of lipotoxicity). Compared to BSA-treated control conditions (Black circles), PA-BSA-treated ventricular myocytes (Red circles) showed significantly increased lipid droplet count (**C**) and size (**D**) and promoted IL-6R expression (**E**–**G**). Data columns are mean ± S.E.M, *n* = 16–37 images. * Statistical significance at *p* < 0.05.

Because of the well-established link between HFD, lipotoxicity, and cardiac inflammation, we next directly tested the role of lipotoxicity in HFD-linked overactivation of cardiomyocyte IL-6 trans-signaling. To mimic the HFD condition in vitro, we challenged ventricular myocytes with PA (a potent inducer of lipotoxicity [8]) and then assessed the expression of IL-6R. Specifically, we found that abnormal lipid droplet count (Figure 10A–C) and size (Figure 10D), classic hallmarks of cardiac lipotoxicity [52,53,54,55,56], could be achieved by treating guinea pig ventricular myocytes with PA (0.5 mM, 2 h), compared to the BSA-alone-treated control. IL-6R expression was increased by 110% following lipid droplet accumulation (Figure 10E–G), supporting a role of lipid droplet remodeling in overactive IL-6 trans-signaling-induced cardiac inflammation.

## 3. Discussion

Obesity is associated with a heightened proinflammatory cytokine response, elevating the risk of adverse arrhythmias [5]. Preventing such pathological effects is a promising direction for therapeutic intervention in the progression of heart disease, and ultimately prevention of VT/SCD risk. In the present study, we found that with our previously developed HFD-induced lipotoxic guinea model [8,9,57] animals displayed significant development of interstitial fibrosis, prolonged QT interval, and high susceptibility to spontaneous ventricular arrhythmias, compared to LFD controls, similar to the changes observed in obese patients. HFD feeding further revealed over-activation of proinflammatory IL-6 signaling in the ventricles of these guinea pigs and was associated with a novel activation and enhanced nuclear translocation of downstream signal transducer and activator of transcription 4 (STAT4), thus providing a unique opportunity to reveal novel cellular proarrhythmic inflammatory mechanisms of obesity VT. Our data showed that IL-6/JAK2 elicits a proarrhythmic electrical response, highlighting the important role of proinflammatory cytokines in the pathogenesis of obese heart. To our knowledge, our study is the first to show a potential role for pathological remodeling of IL-6 signaling, possibly by way of overactivated STAT4 on VT/SCD risk, in obese heart. Our findings further suggest suppression of STAT4 activation may protect against ventricular pathology, representing a novel therapeutic approach for VT/SCD predisposition in obesity.

The Canakinumab Anti-Inflammatory Thrombosis Outcomes Study (CANTOS) showed that canakinumab, a human therapeutic monoclonal antibody targeting interleukin (IL)-1β, significantly reduced major adverse cardiovascular events (MACE) without affecting lipid levels in patients with a history of acute myocardial infarction with elevated C-reactive protein (CRP). However, patients remained at an increased risk of recurring cardiovascular events, particularly among those with the highest levels of IL-6 [58]. Thus, IL-6 signaling could play a fundamental role in cardiovascular prognosis. Intriguingly, in obesity, excess dietary fat in adipose tissue stimulates the release of immunomodulatory cytokines [7,13,14], leading to a state of chronic inflammation in patients. Obese epicardial adipose tissue, a rich local source of IL-6 [59,60,61], may predispose to increased VT/SCD risk [62,63,64], highlighting its utility as an anti-arrhythmic target in a model of obesity VT. Therefore, we focused on IL-6 signaling in this study.

Our study suggested that blockade of IL-6 trans-signaling, in concert with other proinflammatory cytokines, may provide a more favorable clinical outcome than targeting IL-6 trans-signaling alone. For example, IL-1β and IL-18, both of which require the NLRP3 inflammasome for activation [65], can induce the production of copious amounts of IL-6, serving as an amplifier of IL-6 effects. While the effects of IL-1β and TNF-α on cardiac electrophysiology have been studied, the role of IL-18 is under-explored. IL-18 has increased expression in obesity and heart failure [28,29,30], contributes to heart rhythm disorders [66], and VT in mice [67], and our data established the novel finding that IL-18 may heighten cytokine effects on proarrhythmic ion channel functional phenotypes and VT risks.

Channelopathies and ion channel dysfunction remain crucial factors in VT initiation. At the cellular level, normal ventricular cardiac action potential (AP) is defined by Phase 0, due to a large inward sodium current (*I_Na_*), followed by currents due to voltage-gated L-type calcium (Ca) (*I_Ca,L_*) and the Na-Ca exchanger (*I_NCX_*) channels [68]. Repolarization is controlled by a delayed rectifier current (*I_K_*), comprised of rapid (*I_Kr_*) and slow (*I_Ks_*) components. The resting membrane potential is controlled by the inward rectifier *K* current (*I_K1_*) [69]. Thus, decreases in outward currents [8,70,71] or increases in depolarizing mechanisms [72] delay repolarization, resulting in prolongation of the QT interval, which predisposes to fatal VT.

We previously [8,73] demonstrated negative modulation by IL-6 of *I_Kr_* or *I_Ks_* density in guinea pig ventricular myocytes. Other studies showed that IL-6 [74] increases *I_Ca,L_* (which increases calcium load in myocytes) and may be associated with VT [75]. TNF-α has been shown to decrease *I_Kr_* [76], and the transient outward current is also inhibited by IL-1β [77] and IL-18 [67], while IL-1β [78] increases *I_Ca,L_*, together leading to prolongation of the APD. Moreover, we previously [73] showed that the inward sodium current (*I_Na_*) density measured in guinea pig ventricular myocytes was not altered in the presence of acute (40 min) exposure to IL-6. However, we observed marked bradycardia, followed by a complete atrioventricular dissociation, heart block, and asystole in guinea pigs challenged with in vivo applications of IL-6-sIL-6R-IL-18, suggesting an involvement of a decrease in peak *I_Na_.* Although other mechanisms cannot be ruled out, our data support the hypothesis that IL-18, by impacting SCN5A channel biophysics, may contribute to VT/SCD risk by impacting AP upstroke velocity, conduction velocity, and QT interval, highlighting the importance of multi-ion channel analyses that may inform the rational development of safer (reduced cardiotoxic effects), anti-arrhythmic monotherapy and polytherapy approaches for patients.

Here, we further investigated in HFD ventricles the functional expression of two major repolarizing mechanisms, ERG (*I_Kr_*) and KCNQ1-KCNE1 (*I_Ks_*), robustly expressed in humans and guinea pig, and found severe channel modulation manifested as depression of subunit surface expression (ERG1a) and gene (KCNQ1-KCNE1) regulation. Therefore, the inhibitory effect of HFD feeding and/or overactive IL-6-trans-signaling on ERG1 and KCNQ1-KCNE1 channels may occur, at least in part, both at the protein trafficking and transcriptional levels. Notably, our findings demonstrated that HFD shows preferential signaling through STAT4. Accordingly, we show that HFD guinea pigs showed significantly increased pSTAT4/STAT4 expression (62%) compared to LFD-fed controls or pSTAT3/STAT3 (8%). This means that STAT4 may have the potential to significantly regulate the transcription of K channels in obese heart, with implications for clinically actionable targets for prevention of VT/SCD risk. This can be a first step in determining the mechanisms of how major cardiac ion channels may be regulated in the presence of key obesity components (lipotoxicity–cytokines-sympathetic overactivity) and how that affects cardiac function and homeostasis.

Tocilizumab (TCZ, which globally blocks IL-6 activities through IL-6R-α and sIL-6R) [79,80], when used in treating rheumatoid arthritis, is associated with adverse cardiovascular events [81] and an increase in serum cholesterol [82], which increases cardiovascular risk [83,84]. Thus, effective therapeutic interventions for prevention of obesity VT/SCD risk would likely require chronic administration, which would render TCZ ineligible for the long-term treatment of chronic metabolic diseases. However, the potential therapeutic benefits and efficacy of selectively targeting IL-6 trans-signaling (olamkicept) for prevention of VT in obesity are unknown. Interestingly, we found that QT prolongation due to overactivated IL-6 trans-signaling can be prevented with olamkicept, highlighting an emerging and important anti-arrhythmic role for olamkicept in obesity arrhythmias. Notably, olamkicept has shown encouraging results in phase II clinical studies for inflammatory bowel disease [7,85,86,87,88], thus further providing a rationale for refining olamkicept for the development of the next generation of proinflammatory cytokine inhibitors as an antiarrhythmic in the setting of obesity.

### Study Limitations

It is possible that with longer dietary intervention, there may be dispersion in weight differences, and we are currently investigating this possibility as part of a separate study. However, we found that the HFD- and OAD-fed guinea pigs displayed similar change in BW when compared to the LFD controls. We speculated that this may be because the OAD is a variation of the HFD and may still be able to induce weight gain, despite supplementation of the lard (1598/198, HFD vs. OAD) with safflower oil (0/198, HFD vs. OAD). Our focus for this study was not on the increase in body weight but to highlight the importance of differences in dietary fat. OAD-linked inflammatory signaling and its role in arrhythmias is part of an on-going study in our lab. However, as part of this study, we introduced the ability of OAD feeding to rescue HFD linked fibrosis and QT interval, highlighting the beneficial effects of monounsaturated fatty acids. Although not investigated in this study, Oleic acid administration has been shown to lower levels of proinflammatory cytokines, including IL-6 in a model of HFD challenged Wister albino rats [89].

We did not investigate or measure visceral fat in our model. Epicardial adipose tissue (EAT) is a rich local source of IL-6 signaling in obesity [61,90] and a key contributor to a higher-risk of cardiac events in patients [59,91], and therefore likely contributes to cardiac tissue levels. In this regard, EAT has been shown to increase dramatically in guinea pigs, and the amount of EAT is closely correlated to the amount of other types of visceral fat [92]. Therefore, we suspected that EAT is likely to adequately mirror local IL-6 levels defined in pathophysiological conditions, contributing to augmented cellular arrhythmogenesis and increased malignant VT/SCD risk.

## 4. Materials and Methods

### 4.1. Animals, Low-Fat Diet, High-Fat Diet (Palmitic-Acid (PA) Diet), and Oleic-Acid Diet (OAD) Feeding

Guinea pigs (male/female; 300–350 g) were obtained from Charles River Laboratories (Wilmington, MA, USA). The control guinea pigs were fed ad libitum a LFD (Research Diets Inc., New Brunswick, NJ, USA) containing (in kcal%) 10 fat, 70 carbohydrates, 20 protein, and 2300 corn starch. The HFD/PA-diet group was fed a diet (in which most of the soybean was replaced with 1598% lard or 315 kcal% palm oil) containing 45%/10% of kilocalories from fat, 35%/70% from carbohydrates, and 20% from protein. The monounsaturated OAD group was challenged with a diet in which the soybean was substituted for 198% safflower oil and contained (in kcal%) 45% fat, 35% carbohydrates, and 20% protein. The HFD/PA-rich diet contained saturated and unsaturated free fatty acids (FFAs), which provided 31.6%/48.4% and 35.5%/36.8% of the fat-derived calories, while the LFD provided 23.5% and 29.7% and OAD provided 46.2% and 41.4%. Guinea pig groups were challenged with specific diets for a duration of 100 days (~14 weeks).

### 4.2. Electrocardiogram (ECG)

Surface ECG was recorded in slightly anesthetized guinea pigs using a Dual Animal BioAmp amplifier PowerLab (LabChart 8/s, AD instruments, Colorado Springs, CO, USA) and analysis system (LabChart v8.1.2, AD instruments, Colorado Springs, CO, USA). Guinea pigs were placed on a warm pad and subjected to anesthetic inhalation, using a table-top isoflurane (3–5%) vaporizer (Harvard Apparatus, Holliston, MA, USA). A cone mask was used to maintain anesthesia with 1–2% isoflurane (mix of isoflurane and 700 mL O_2_/min). Anesthesia depth from isoflurane was monitored by respiratory rate and toe pinch response. Electrodes were positioned on the sole of each guinea pig foot. After a 10 min stabilization period, 2 boluses of cytokines, and coumermycin were injected at 15 min intervals. Electrical signals were recorded at 1200 Hz, stored on a computer hard disk, and analyzed off-line using the average of five representative consecutive beats. Tracings were analyzed and calculated for QT_c_ interval by Bazett’s formula where QTc = QT/√RR.

### 4.3. Estimation of Interstitial Fibrosis in Ventricular Tissue Slices from Guinea Pig

Tissue was fixed in 4% paraformaldehyde and then dehydrated with the standard gradient of 70% to 100% ethanol concentration and then cleared with Citrisolv prior to paraffinization. Tissue was sectioned with a HistoCore BIOCUT Microtome (Leica Biosystems, Wetzlar, Germany) and samples were adhered to slides and stained using an automated Masson’s trichrome protocol on a Dako Artisan Link Pro (Agilent, Santa Clara, CA, USA). After initial dehydration of the samples, all sectioning and processing was performed at Associated Regional and University Pathologists Inc. (ARUP, Salt Lake City, UT, USA). Slides were then imaged on an EVOS XL Core microscope (Invitrogen, Carlsbad, CA, USA) with a 20× objective at room temperature (20–25 °C) and quantification of fibrotic area was performed using MATLAB R2023a (RRID:SCR_001622).

### 4.4. Enzyme-Linked Immunosorbent (ELISA) Assay

Guinea pig serum and cardiac tissue cytokine levels were measured using ELISA kits (R&D systems, Minneapolis, MN, USA) according to the manufacturer’s instructions.

### 4.5. Preparation of Bovine Serum Albumin (BSA)-Conjugated FFA Solutions

PA stock solution was prepared as previously described [9]. Briefly, fatty-acid-free bovine serum albumin (BSA, Roche, Sigma-Aldrich, Inc. St. Louis, MO, USA) (20%) was dissolved in Dulbecco Phosphate Buffered Saline (DPBS) and filtered to sterilize. The saturated PA (Sigma-Aldrich, St. Louis, MO, USA) was dissolved in ethanol to make a 0.2 M fatty acid (FA) stock solution. BSA (20%) and PA (0.2 M) were mixed in a 20:1 volumetric ratio. FA stock solutions (~10 mM) were directly added to M199 cardiomyocyte culture medium to a final concentration of 0.5–1 mM.

### 4.6. Guinea Pig Ventricular Myocyte Isolation

Adult male and female Hartley guinea pigs were deeply anesthetized with isoflurane in accordance with the guidelines of the Declaration of Helsinki and approved by the Institutional Review Board (or Ethics Committee) of the University of Utah (Protocol #: 21-09006) Animal Care and Use Committees and conforming to NIH guidelines. Primary myocyte isolation procedures have been previously described [8,71].

### 4.7. LipidSpot Lipid Droplet Staining

Guinea pig ventricular myocytes were pre-exposed (2 h) to either BSA alone or PA-BSA (0.5 mM) and were subsequently incubated with a solution containing paraformaldehyde (4%) supplemented with glucose (4%) for 10 min at room temperature (23–25 °C). Fixed ventricular myocytes were labeled with Lipid Spot 610 (#70069; Biotium, Fremont, CA, USA) to stain aggregated lipid droplets and excited with a 561 nm laser, and emission was collected at 592–638 nm. Immunohistochemistry was performed using a 1:100 dilution of anti-IL6Ra (H-7) mouse mAb (#sc-373708, Santa Cruz Biotechnology, Dallas, TX, USA, RRID:AB_10947248) and a 1:100 dilution of anti-IL-6 (1) mouse mAb (#sc-130326, Santa Cruz Biotechnology, Dallas, TX, USA, RRID:AB_2127744). Donkey anti-Mouse IgG (H + L) Highly Cross-Adsorbed Secondary Antibody, Alexa Fluor 488 (#A-21202, ThermoFisher Scientific, Waltham, MA, USA, RRID:AB_141607) was used and excited with a 488 nm laser and emission was collected at 491–610 nm. Two dimensional images were acquired on a Leica SP8 confocal microscope using a GaAsP-HyD detector and a 40× or 63× oil immersion lens (numerical aperture 1.2) with a 0.1 × 0.1 μm pixel size at room temperature. All samples were imaged with identical imaging parameters. Sequential framing approaches were used to avoid simultaneous excitation of fluorophores and minimize cross-talk. Images were processed for noise reduction and background correction, then visualized with the same intensity ranges for comparison. Lipid droplet size and count were quantified as described previously [93] using MATLAB R2023a (RRID:SCR_001622).

### 4.8. Quantitative Reverse Transcription Polymerase Chain Reaction (qRT-PCR)

Total RNA from guinea pig ventricular tissue was extracted with RNeasy^®^ Plus Mini Kits (74134, Qiagen, Hilden, Germany). The quantity, purity, and integrity of RNA and cDNA samples were determined by spectrophotometry (NanoDrop Lite Plus, Thermo Fisher Scientific, Madison, WI, USA). cDNA synthesis was carried out using an iScript cDNA Synthesis Kit (1708891, BioRad, Hercules, CA, USA). All assays were performed according to the manufacturer’s supplied protocol. qRT-PCR assays were performed using PowerUpTM SYBR™ Green Master Mix (A25742, Applied Biosystems, Vilnius, Lithuania) and a CFX Opus 96 Real-Time PCR System (#12011319; Bio-Rad, Hercules, CA, USA). All primer sequences for genes of interest are listed in Table 3. Relative expression values and fold changes were calculated using the ΔΔCt analysis method relative to GAPDH (endogenous control) and LFD as the control sample, and are presented as mean ± SEM. Primers were designed to span exon–exon regions, to avoid amplification of genomic DNA, and primer specificity was verified by blasting all sequences using the NCBI Primer-BLAST tool (http://www.ncbi.nlm.nih.gov/tools/primer-blast/; RRID:SCR_003095 (accessed on 10 July 2023).

### 4.9. Immunofluorescence, Confocal Imaging, and Image Analysis

Guinea pig ventricular tissues were fixed in 4% paraformaldehyde and sectioned using a vibratome (#VT1200, Leica, Deer Park, IL, USA) at a 20 μm thickness. Immunohistochemistry was performed using the following primary antibodies: 1:100 dilution of anti-Phospho-STAT3 (Tyr705) (D3A7) XP^®^ rabbit mAb (#9145 Cell Signaling, Danvers, MA, USA, RRID:AB_2491009); 1:100 dilution of anti-STAT3 (F-2) mouse mAb (#sc-8019, Santa Cruz Biotechnology, Dallas, TX, USA, RRID:AB_628293); a 1:100 dilution of anti-IL6Ra (H-7) mouse mAb (#sc-373708, Santa Cruz Biotechnology, Dallas, TX, USA, RRID:AB_10947248); 1:100 dilution of anti-IL-6 (1) mouse mAb (#sc-130326, Santa Cruz Biotechnology, Dallas, TX, USA, RRID:AB_2127744); 1:200 dilution of anti-hERG1b rabbit pAb (#ALX-215-051-R100, Enzo, Farmingdale, NY, USA, RRID:AB_2051587); 1:200 dilution of anti-hERG1a (NT) rabbit (#ALX-215-050-R100, Enzo, Farmingdale, NY, USA, RRID:AB_2051586); 1:50 dilution of anti-phospho-Stat4 (Tyr693) rabbit pAb (#5267, Cell Signaling, Danvers, MA, USA, RRID:AB_10545446); 1:50 dilution of anti-Stat4 (C46B10) rabbit pAb (#2653, Cell Signaling, Danvers, MA, USA, RRID:AB_22551556); 1:50 dilution of anti-IL-18 (D2F3B) rabbit pAb (#54943, Cell Signaling, Danvers, MA, USA, RRID:AB_2909592); and 1:100 dilution of anti-TGFβ rabbit pAb (#21898-1-AP, ProteinTech, Rosemont, IL, USA, RRID:AB_2811115). Donkey anti-Rabbit IgG (H + L) Highly Cross-Adsorbed Secondary Antibody, Alexa Fluor™ 647 (#A-31573 ThermoFisher Scientific, Waltham, MA, USA, RRID:AB_2536183) or Donkey anti-Mouse IgG (H + L) Highly Cross-Adsorbed Secondary Antibody, Alexa Fluor™ 647 (#31571, ThermoFisher Scientific, Waltham, MA, USA, RRID:AB_162542) were used at a dilution of 2 μg/mL. Wheat germ agglutinin (WGA) conjugated to Alexa Fluor 488 (#W11261, ThermoFisher Scientific, Waltham, MA, USA) and DAPI (#D1306, ThermoFisher Scientific, Waltham, MA, USA) were used following the instructions of the manufacturer. Two-dimensional images were acquired on a Leica SP8 confocal microscope using a GaAsP-HyD detector and a 40× oil immersion lens (numerical aperture 1.2) with a 0.1 × 0.1 μm pixel size at room temperature. DAPI was excited with a 405 nm laser and emission collected at 410–550 nm. WGA was excited with a 488 nm laser and emission collected at 491–610 nm. Alexa Fluor 647 conjugated antibody was excited with a 633 nm laser and emission collected at 638–775 nm. All samples were imaged with identical imaging parameters, including pinhole size (1 Airy unit). Sequential framing was used to limit simultaneous excitation of fluorophores and minimize cross-talk. Images were processed for noise reduction and background correction, then visualized with the same intensity ranges for comparison. Fluorescence colocalization was quantified in MATLAB R2023a (RRID:SCR_001622).

### 4.10. Statistical Analyses

Data are reported as means ± S.E.M. Statistical differences were determined using two-tailed unpaired *t* test for comparisons between groups and considered significant at *p* < 0.05. For the imaging, cytokine ELISA and mRNA expression experiments the comparisons were LFD vs. HFD or OAD, as well as control (BSA) vs. PA-BSA. For all ECG experiments, the comparison was QTc Basal vs. QTc post-intervention. All experiments were blinded to experimenters and those performing the analyses. Data points were discarded as outliers only if they were greater than three standard deviations from the group.

## 5. Conclusions

To our knowledge, we are not aware of any studies that have mechanistically established the link between IL-6 trans-signaling, ion channel regulation, and VT in HFD guinea pigs that show high vulnerability to arrhythmias. Specifically, our data revealed over-activation of IL-6 signaling is associated with a novel activation and enhanced the nuclear translocation of downstream signal transducer and activator of transcription 4 (STAT4). We further found a larger accumulation of interstitial fibrosis, increased TGF-β expression in HFD hearts, and direct stimulation of JAK2-induced arrhythmias. At the cellular level, our data showed that cardiac lipotoxicity overactivated IL-6 trans-signaling and this occurred by way of lipid droplet accumulation. Overall, the present study is the first to demonstrate that pathologically overactive IL-6 trans-signaling and JAK2 activation lead to dramatic pathology and are directly associated with VT. IL-6 and IL-18 are emerging as relevant cytokines involved in the inflammatory process, with severe ventricular electrophysiological consequences [8,67].

### Clinical Perspectives

IL-6 is a pleiotropic cytokine (downstream of IL-1β action) [13] and is a powerful predictor of the severity of heart disease [6]. Crucially, IL-6 trans-signaling inhibition has the potential to enhance the efficacy and even exceed the beneficial effects (anti-arrhythmic) of anti-inflammatory drugs currently in clinical trials. While anti-inflammatory (including non-steroidal anti-inflammatory [94,95], and corticosteroids [96,97]) drugs have shown beneficial anti-arrhythmic properties in experimental atrial fibrillation (AF) [98], all have shown an increased risk of AF in patients, likely because our knowledge of cytokine mechanisms is incomplete. Understanding of the link between IL-6-STAT4 and obesity VT may have broad clinical implications for many cardiac rhythm disorders. Our findings are consistent with other studies that demonstrated that acute inflammation induces electrical remodeling, and these effects can be prevented with the anti-inflammatory agent colchicine, which ultimately leads to decreases in the recurrence of AF [99,100,101,102,103]. Thus, future efforts that interrogate whether therapeutic manipulation of IL-6-STAT4 efficiently prevents proarrhythmic signatures (ion channel function, APD phenotypes, QT prolongation, VT/SCD vulnerability) in diseased HFD/obese ventricular tissues/myocytes, thus counteracting the redundancy of cytokines and undesired side-effects elicited by breaching the essential homeostatic role of classical IL-6 signaling, are likely to be rewarded with novel therapeutic perspectives and beneficial translational implications in patients.

## Figures and Tables

**Table 1:** QT_c_ interval measured in guinea pigs challenged with different dietary interventions.

Conditions	QT_c_ (ms) (Basal)	QT_c_ (ms) (Post-Intervention)	*p* Value	ΔQT_c_ (ms)	*n*
Low-fat diet (LFD)	279.8 ± 15.2	269.4 ± 18.1	0.665	−10.5 ± 4.51	8
High-fat diet (HFD)	260.4 ± 15.1	323.9 ± 15.2 *	0.0018	63.32 ± 10.9	15
Oleic acid diet (OAD)	294.7 ± 6.14	307.8 ± 5.58	0.137	13.1 ± 8.09	8

Data are means ± S.E.M. * *p* < 0.05 compared to corresponding basal controls, two-tailed paired Student’s *t* test.

**Table 2:** Effect of overactive pro-inflammatory cytokine and *I_Ks_* inhibition on QT_c_ interval measured in guinea pigs.

Conditions	QT_c_ (ms) (Basal)	QT_c_ (ms) (Post-Intervention)	*p* Value	ΔQT_c_ (ms)	*n*
Vehicle	297.3 ± 2.54	302.7 ± 3.94	0.298	5.42 ± 1.45	4
IL-6-sIL-6R	289.3 ± 0.72	310.9 ± 4.22 *	0.003	21.66 ± 6.12	5
hyperIL-6 (hIL-6)	299.7 ± 2.61	333.9 ± 8.63 *	0.004	34.8 ± 8.15	8
hIL-6 + Olamkicept	292.9 ± 5.47	293.8 ± 4.24	0.899	0.93 ± 8.30	3
Coumermycin	299.1 ± 5.40	337.5 ± 10.7 *	0.01	38.4 ± 10.9	7
IL-6-sIL-6R + IL-18	292.3 ± 10.7	319.2 ± 10.7 *	0.0004	26.95 ± 3.98	6
Chromanol293B	308.6 ± 4.24	327.9 ± 5.68 *	0.018	19.23 ± 3.35	8
Chromanol293B + ISO- 10 min	308.6 ± 4.24	342.5 ± 7.69 *	0.004	33.0 ± 10.4	6
Chromanol293B + ISO + Cytomix- 10 min	308.6 ± 4.24	363.6 ± 6.48 *	0.0036	54.2 ± 11.02	3
Chromanol293B + ISO + Cytomix- 30 min	308.6 ± 4.24	377.8 ± 15.3 *	0.03	68.4 ± 17.8	3

Data are means ± S.E.M. * *p* < 0.05 compared to corresponding basal controls, two-tailed paired Student’s *t* test.

**Table 3:** List and sequence of primers used in this manuscript for qPCR amplification.

Gene	Sequence (5′- > 3′)	Gene ID
IL-6R	sense GGGTCGGGCTTCAAGATGTTA antisense AACGGTGCCTGTATTCTGGG	100730490
JAK2	sense CTTAGATTACGCCGCCCAGC antisense TGTGCCGGTATGACCCTCTA	100722908
KCNQ1	sense GCTGTTCTCTGAGGGTCTTCCA antisense CCATCCACCCTGAACTCTTTCT	100379230
KCNE1	sense TCCCAGGAAAACTGTCAGCTC antisense CGGTTCTGAGGAAGCGGATT	100135562

## Data Availability

All the relevant data are included within the paper itself.

## Abbreviations

ERG	Ether-à-go-go related gene
CVC	Cranial vena cava
FFA	Free fatty acid
QT_c_	Heart rate corrected QT interval
HFD	High-fat diet
hERG	Human ether-à-go-go related gene
hIL-6	Hyper-IL-6
IL-6	Interleukin-6
IL-1β	Interleukin-1 beta
JAK2	Janus kinase 2
LFD	Low-fat diet
OAD	Oleic acid diet
PA	Palmitic acid
I*_Kr_*	Rapidly activating delayed rectifier K current
I*_Ks_*	Slowly activating delayed rectifier K current
STAT4	Signal transducer and activator of transcription 4
sIL-6	Soluble interleukin-6 receptor
TGF-β	Transforming growth factor beta
TNF-α	Tumor necrosis factor alpha
